# “Guarding their practice”: a descriptive study of Canadian nursing policies and education related to medical cannabis

**DOI:** 10.1186/s12912-019-0390-7

**Published:** 2019-12-09

**Authors:** Lynda G. Balneaves, Abeer A. Alraja

**Affiliations:** College of Nursing, Rady Faculty of Health Sciences, University of Manitoba, 89 Curry Place, Winnipeg, Manitoba R3T 2N2 Canada

**Keywords:** Marijuana, Nurse practitioners, Advanced nursing practice, Scope of practice, Professional regulation, Nursing legislation, Canada

## Abstract

**Background:**

In Canada, federal regulations allow Nurse Practitioners (NPs) to authorize medical cannabis. Nursing regulatory bodies, however, have been hesitant to include medical cannabis within NPs’ scope of practice. As the interest in cannabis increases, NPs have the potential to play a pivotal role in promoting the safe and appropriate use of cannabis. This study aimed to: summarize nursing policies in Canada related to medical cannabis; explore the perspective of nursing regulatory bodies regarding practice and policy issues related to medical cannabis; and examine the inclusion of medical cannabis content within Canadian NP curricula.

**Methods:**

A descriptive study was conducted that comprised three phases. The first phase reviewed nursing regulatory bodies’ existing policies related to medical cannabis. In the second phase, practice consultants from nursing regulatory bodies were interviewed regarding policies and practices issues related to medical cannabis. The interviews were analyzed using thematic analysis. The third phase was a national survey of NP program coordinators regarding inclusion of cannabis in curricula. Descriptive statistics summarized survey responses.

**Results:**

Of the 12 nursing regulatory bodies in Canada, only 7 had policies or statements related to cannabis, with only Ontario allowing NPs to authorize medical cannabis. There was confusion among practice consultants regarding the role of nurses in the administration of medical cannabis and several barriers were identified regarding nursing engagement in care related to medical cannabis, including lack of knowledge and clinical guidelines. 60% of NP programs included cannabis in their curricula, however, less than half addressed the risks and benefits of medical cannabis and dosing and administration protocols. Limited faculty expertise was a barrier to including cannabis content in NP curricula.

**Conclusion:**

Nursing regulatory bodies must be proactive in developing policies and educational resources that will support nurses in providing safe and informed care related to cannabis. To ensure patients using medical cannabis receive consistent and safe care from nurses, harmonized regulations and policies are needed across all jurisdictions. Education programs must also provide updated knowledge and training for both registered nurses and NPs that will support them in providing non-judgemental and evidence-based care to the growing number of individuals using cannabis.

## Background

Medical cannabis is a growing phenomenon in Canada and is anticipated to increase following the legalization of non-medical cannabis. In 2001, Canada was one of the first countries to regulate medical cannabis, establishing the Marihuana Medical Access Regulations (MMAR). Since that time, a series of federal regulations have come into effect, including the Medical Marihuana Program Regulations (MMPR) and the Access to Cannabis for Medical Purposes Regulations (ACMPR), which have steadily expanded Canadians’ legal access to medical cannabis through an increasing number of licensed producers, as well as the extension of authorization rights to physicians and nurse practitioners (NPs) [1]. In addition, patients have continued to petition the Canadian government for the legal right to either grow their own cannabis for therapeutic purposes or designate someone to grow for them. By the end of September 2018, just before the legalization of non-medical cannabis, 342,103 individuals were registered under the ACMPR [2].

On October 17, 2018, the Cannabis Act and Regulations came into effect that legalized non-medical cannabis nationwide [3]. The goals of this legislation were to eliminate the harms associated with the illicit market, to create a regulated and safe supply of non-medical cannabis, and to limit access to non-medical cannabis by youth [3]. In addition, this legislation aimed to consolidate regulations regarding cannabis for medical and non-medical purposes, with a slightly modified version of the ACMPR embedded within the Cannabis Regulations. While there is no difference between medical and non-medical cannabis in terms of type and quality of product (i.e., dried cannabis and oils), the regulations related to eligible age of consumption, the amount of cannabis allowed in public, and where medical versus non-medical cannabis can be consumed differ between medical and non-medical cannabis federally as well as across numerous provincial/territorial jurisdictions.

The legalization of non-medical cannabis is anticipated to increase the number of Canadians using medical cannabis, especially among those who have experienced challenges in gaining authorization from their healthcare provider [4]. The stigma associated with cannabis use and the belief by clinicians that it is only a recreational substance with a high potential for abuse has been a significant barrier experienced by patients to medical cannabis authorization [4]. In addition, lack of knowledge and clear practice guidelines have been identified by physicians as preventing them from authorizing medical cannabis [5]. As such, a substantial number of Canadians have been forced to access medical cannabis from illegal sources and without authorization from their healthcare providers [6]. Following legalization, these individuals can now access cannabis for therapeutic purposes through the non-medical retail market.

The inclusion of NPs as healthcare providers able to authorize medical cannabis was initially presented as a way to facilitate access, particularly for patients without access to a primary care physician or specialist. However, several nursing regulatory bodies in Canada still do not permit NPs to authorize herbal cannabis. This is despite all Canadian provinces and territories allowing NPs to prescribe controlled substances, such as opioids. Under the new cannabis regulations, RNs and LPNs are also allowed to possess, distribute and directly or indirectly assist in the administration of medical cannabis to an authorized individual; however, nurses’ scope of practice related to medical cannabis differs across jurisdictions as well as clinical institutions. Moreover, at the time of the study and before the Cannabis Act and Regulations were enacted, the Canadian Nurses Protective Society (CNPS) had recommended:

In light of the explicit authorization given to physicians and NPs to administer the substance, the corresponding lack of legislative authorization given to RNs to do so and the use of the phrase “providing assistance in the administration” of cannabis, it is recommended that RNs do not directly administer cannabis to patients at this time, even when they are provided with a valid medical document issued by an NP or a physician ([7], p. 30).

### Nurses’ information/education needs regarding medical cannabis

A national survey was conducted to assess NPs’ knowledge, experience, barriers, and attitudes toward medical cannabis [8]. The results of this survey indicated NPs lacked knowledge about medical cannabis and the majority of participants ranked their need for cannabis education to be either strong or very strong [8]. To date, there has been limited education available to nurses related to medical cannabis, with the exception of online courses offered by such organizations as the American Cannabis Nurses Association and the Canadian Nurses Association. Both of these courses are available for a fee and provide foundational knowledge about cannabis and its effects.

As the interest in cannabis as a therapeutic and recreational agent increases in Canada, NPs will play a pivotal role in promoting the safe, effective and appropriate use of medical and non-medical cannabis. To understand the current policy context, the existing barriers and facilitators to medical cannabis being included within NPs’ scope of practice, and the education needs of NPs related to cannabis, an exploration of national and regional nursing policies and NP education programs was needed. The aims of this study were: 1) summarize existing nursing policies in Canada related to medical cannabis; 2) explore the perspectives and experiences of nursing practice consultants from provincial/territorial regulatory bodies in Canada regarding the current practice and policy issues related to medical cannabis; and 3) examine the extent of medical cannabis content within Canadian NP education programs, as well as the perceived barriers to including medical cannabis within NP curricula and future plans related to medical cannabis education.

## Methods

In this multi-phase descriptive study, the first phase focused on identifying nursing policies, including position statements and practice standards, related to medical cannabis. A search of nursing regulatory bodies’ websites was undertaken using the terms “cannabis” and “marijuana/marihuana”. This search was undertaken in May 2017 and updated in August 2018.

Telephone interviews were then conducted from September 2017 to April 2018 with practice consultants from nursing regulatory colleges and associations across Canada regarding existing and pending nursing policies related to medical cannabis. These individuals were identified during the search of regulatory websites and through phone calls to each organization. Eligibility criteria included being over 18 years of age, able to read/speak English, and employed as a practice consultant by a provincial/territorial nursing regulatory body in Canada. A letter of invitation was sent via email, with a maximum of three follow-up email reminders sent to encourage participation in the study. During the interviews, participants were asked about current and future nursing policies related to medical cannabis, potential challenges and facilitators faced by nurses related to cannabis, and the perceived impact of cannabis legalization on the nursing profession (see Additional file 1 for interview guide). The interviews were analyzed using qualitative thematic analysis [9]; interviews were read and re-read, and line-by-line coding was completed by both authors (LGB/AAA). The initial codes were reviewed and collapsed into major themes and sub-themes based on the interview questions. Final coding was conducted by both authors (LGB/AAA) and any disagreements were resolved through a consensus process. An audit trail of coding decisions and meetings between the authors was kept.

Lastly, a national online survey of NP program coordinators identified from university websites was conducted from September to December 2017. Eligibility criteria included being over 18 years of age, able to read/speak English, and employed as a coordinator of a NP education program in Canada. Letters of invitation were sent via email to eligible participants, with three email reminders sent each of the following weeks.

The investigator-developed survey (see Additional file 1) explored the presence of medical cannabis content in NP curricula, including which courses covered medical cannabis, barriers to inclusion of medical cannabis content in curricula, and future plans regarding addressing medical cannabis within the NP program. The online surveys were distributed via a survey software program (Qualtrics®) and two reminder emails were sent. Descriptive statistics were used to summarize demographic information and responses to the survey items. Data were entered and analyzed using Microsoft Excel® (Redmond, USA).

The study received ethical approval from the University of Manitoba’s Education and Nursing Research Ethics Board (Protocol #E2017:065 (HS20993)). Written informed consent was received from the practice consultants and implied consent was received from NP coordinators who completed the online survey.

## Results

### Medical cannabis policy review

Just prior to the legalization of non-medical cannabis in Canada, 58.3% (7/12) of the identified provincial/territorial nursing regulatory bodies had policies or statements related to cannabis. This included practice statements and guidelines, position papers, and frequently asked questions (FAQs) that addressed nursing practice issues related to cannabis. The majority of regulatory bodies with policies on medical cannabis addressed nurses’ role related to the administration of medical cannabis, including information about possession, distribution, and administration within specified locations (i.e., hospitals). Not all regulatory bodies, however, made a distinction between nurses assisting with administration versus directly administering medical cannabis to authorized patients. In addition, nurses were cautioned to check with their employers’ policies prior to engaging in care related to medical cannabis. In many cases, cannabis was mentioned within the regulatory bodies’ documents related to controlled substances, in which it was delineated as a substance that NPs were not allowed to authorize. The only exception, at the time of the study, was Ontario, which had a policy that allowed NPs to authorize medical cannabis to eligible patients, and Nova Scotia, which allowed pharmaceutical forms of cannabis to be prescribed by NPs.

### Practice consultant interviews

In Canada, there are a total of 12 nursing regulatory bodies representing 11 provinces and 3 territories. A total of 8 participants from 7 nursing regulatory bodies responded and agreed to be interviewed regarding their regulations, policies and standards, as well as the practice issues they were currently experiencing, related to medical cannabis. There was representation from both Western (*n* = 3) and Eastern Canada (*n* = 3), as well as Northern Canada (*n* = 1). Of the 7 regulatory bodies represented, 5 had policy statements on medical cannabis (71.4%). Practice consultants from the remaining five regulatory bodies either indicated that participating in a research study was not considered the purview of a regulatory body (*n* = 4) or no response was received despite two follow-up invitations (*n* = 1).

#### Current regulations related to nursing and medical cannabis

Out of the 7 regulatory bodies included in this study, none reported at the time of the study developing regulations that permitted NPs to authorize medical cannabis within their region. With regards to the administration of medical cannabis, there was a great deal of confusion and trepidation among practice consultants regarding the ACMPR and the role of registered nurses. Several consultants spoke of the “ambiguity” of the language in the federal regulations regarding whether registered and licensed practical nurses could assist patients in self-administration versus directly administer cannabis to patients unable to do so themselves. Others interpreted the federal regulations as not authorizing nurses to directly administer medical cannabis to patients. In addition, several consultants raised concerns regarding the specificity of the ACMPR in limiting nursing practice related to medical cannabis to hospital settings only, excluding nurses working in community settings: “*Our direction now is that nurses can only directly administer [medical cannabis] in a hospital or a long-term care setting and cannot directly administer in a home setting.”* All consultants, however, spoke of the need for nurses to be competent and have the necessary knowledge, skills and training to provide safe care related to medical cannabis. To this end, some of the regulatory bodies referred nurses interested in incorporating medical cannabis into their practice to general medication administration standards. Further, registered nurses were encouraged to consult their employers regarding any pertinent policies related to medical cannabis.

According to the consultants interviewed, the 2017 CNPS document was influential in how their regulatory body approached the issue of medical cannabis. Several of the colleges consulted with the CNPS in crafting their response to nurses inquiring about the inclusion of medical cannabis in their clinical practice. Nurses were also encouraged to individually consult with CNPS about their practice concerns related to cannabis. The ambiguity in the federal regulations led several of the regulatory bodies to encourage the CNPS to advocate for changes to the federal regulations regarding nurses’ scope of practice in relation to medical cannabis.

*We’ve advocated to CNPS that because community nursing is really the heart and soul of how we can care for our community, this is what’s actually the barrier for us in making sure that our nurses are protected and able to care for patients appropriately.*

#### Future plans regarding medical cannabis regulations

The majority of practice consultants indicated their regulatory bodies were waiting for the legalization of non-medical cannabis before moving forward with any changes to their current nursing regulations and standards related to medical cannabis.

*It’s under review and I know that there will be changes to federal legislation, but we don’t know what that is so we don’t really know what the implications could be to our practice standards yet.*

In the meantime, a few regulatory bodies were moving forward with education and practice initiatives. This included the development of a regulatory framework for the authorization of medical cannabis by NPs, and a “practice direction” outlining the standards that must be met in order for NPs to participate in the authorization of medical cannabis [10].

#### Barriers to nurses’ engagement in care involving medical cannabis

Beyond the lack of clear federal regulations, several barriers were identified that prevented nurses from being more actively engaged in care related to medical cannabis. Foremost, the absence of practice guidelines regarding dose and administration was perceived to pose a significant challenge to NPs being able to authorize medical cannabis. Several consultants spoke about “prescriptions”, comparing medical cannabis to pharmaceutical medication, and how it violated standard medication administration principles:

*With medical cannabis, there is no established best practice guidelines, there’s no dosage for a registered nurse or a nurse practitioner if they were writing it as a prescription, there’s no dosage and it hasn’t been approved by Health Canada.*

Personal and structural barriers were also identified with regards to nurses’ clinical engagement with medical cannabis. Some nurses were perceived as holding values or beliefs that could lead to a moral dilemma in assisting patients using medical cannabis. As one consultant shared: *“I think there are issues around biases and nurses wanting to or not wanting to be involved in administering cannabis, for personal reasons or viewpoints.”* In addition, several consultants indicated that many healthcare organizations currently lack policies regarding the use of medical cannabis in their facilities, leaving nurses feeling confused and unsupported regarding how to address cannabis as part of their practice. Moreover, the occupational health and safety issues related to patients using inhaled forms of cannabis, especially in home care settings, and exposing nurses to second-hand smoke further complicated the potential role of nurses in distributing and administering cannabis.

#### Facilitators of nurses’ engagement with medical cannabis

Increasing nurses’ competency was perceived by many consultants as being integral to the inclusion of medical cannabis within nurses’ scope of practice:

*If we had any practitioners that were going to be engaging with this [medical cannabis], we would establish parameters for some kind of education or training as an expectation of regulation that we would want people to undertake before they engage in that practice.*

To achieve this competency, the consultants were supportive of the inclusion of cannabis within NP training programs as well as the development of continuing education for those nurses already in practice. In some regions, funding was available for nurses interested in pursuing medical cannabis education. Many of the consultants, however, were very clear that from the perspective of a regulatory body, each nurse had to self-determine what knowledge, skills, abilities, and competencies were required in order to safely provide medical cannabis care.

Given the aforementioned barriers and policy challenges, it was not surprising that having consistent and harmonized medical cannabis regulations and policies at the federal, provincial/territorial and institutional level were identified as being a key facilitator to supporting nurses’ engagement with medical cannabis at point of care. Such policies would ensure nurses were legally protected in handling, distributing and administering medical cannabis, or as one consultant framed it, “*guard their practice*.”

#### Practice issues related to cannabis

Several practice issues related to cannabis were raised by the consultants. Following legalization, there was a belief that nurses would need to be able to assess for problematic use within general and disease-specific populations (e.g., mental health). In turn, nurses would need to become more informed about harm reduction strategies specific to cannabis. Knowledge of the indications, contraindication and adverse effects, as well as appropriate storage and disposal, of medical cannabis was also described as being an essential part of future nursing care.

A final point raised by several consultants was the use of cannabis by nurses themselves. Consultants agreed that nurses must self-determine their own fitness to practice following the consumption of cannabis as part of their accountability as a nurse. Those practicing impaired would be subject to sanctions and those suspecting impairment would have a duty to report such behaviour. As one consultant shared:

*There is an assumption that they have to be fit to practice … I think everyone realizes that they are held to being accountable for their own practice and their own decisions and their own fitness to practice in those choice. Those people that are making choices that might impact their fitness to practice will have to accept the consequence if that is being reported. We have to trust that people are going to do the right thing.*

Several consultants, however, pointed out how “tough” determining fitness to practice is in relation to cannabis consumption, requiring not only blood or urine tests, but also measures of cognitive and behavioural functioning that are not easily measured. Another consultant pointed out the complexity of the fitness to practice concept in the context of medical cannabis use:

*If they’re using it for a medical reason, have they actually become more fit to practice because it’s helped with that symptom that they’re having and now that that symptom is gone, they are more fit to practice and the cognitive side effect or other side effects are limited?*

### National survey of nurse practitioner education programs

A total of 28 NP program coordinators were identified and invited to participate in the online survey. Ten respondents completed the survey (35.7% response rate). The coordinators were geographically located throughout Canada, from British Columbia (*n* = 1), Ontario (*n* = 3), Atlantic provinces (*n* = 2), and the Prairies (*n* = 3). The majority of the NP programs had been operational for more than 10 years (60%) and half reported having 30–60 NP students/year enrolled. In terms of the types of NP programs offered, family practice and primary care comprised the largest proportion. In Ontario, NP education has been standardized across all NP programs in the province. See Table 1 for more details.

**Table 1:** Participant Demographic Characteristics (*n* = 10)

Characteristics	Frequency (%)
Geographical Location	
BC	1 (10.0)
Prairies	3 (30.0)
ON	3 (30.0)
Atlantic	2 (20.0)
Did not respond	1 (10.0)
Age of NP Program	
5 < 10 years	1 (10.0)
> 10 years	6 (60.0)
Did not respond	3 (30.0)
Number of Students in Program	
0 < 30	1 (10.0)
30 < 60	5 (50.0)
> 60	1 (10.0)
Did not respond	3 (30.0)
Type of NP Program^a^	
Family Practice	4 (40.0)
Primary Care	4 (40.0)
Adult	3 (30.0)
Pediatric	1 (10.0)
Post-graduate certificate	2 (20.0)
Did not respond	1 (10.0)

^a^Respondents could identify more than one type of NP Program

### Content areas specific to medical cannabis

More than half of the respondents indicated that their NP program included various topics specific to medical cannabis (6 out of 10), including mechanism of action (*n* = 4) and the Canadian laws and regulations surrounding medical cannabis (*n* = 4). Half of the programs (*n* = 3) with medical cannabis content also reviewed the therapeutic benefits and risks associated with cannabis. Only one program included content on medical cannabis dosing and treatment plans. See Table 2 for further details.

**Table 2:** Content Areas Specific to Medical Cannabis (*n* = 6)

Items	Frequency^a^ (%)
Dosing and creating effective treatment plans for patients using medical cannabis	1 (16.7)
Similarities and differences between dried cannabis, other forms of cannabis products, and prescription cannabinoid medications	2 (33.3)
Health Canada’s Access to Cannabis for Medical Purposes Regulations Program	2 (33.3)
Laws and regulations surrounding the medical use of cannabis in Canada	4 (66.7)
Safety, warning signs and precautions for patients using medical cannabis	2 (33.3)
Alternative routes of administration of medical cannabis	2 (33.3)
Mechanism of action of cannabis (endocannabinoid system)	4 (66.7)
Potential risks of using cannabis for medical purposes	3 (50.0)
Potential therapeutic uses for cannabis	3 (50.0)
Other (please specify)	1 (16.7)

^a^Respondents could select more than one option

### Barriers to including medical cannabis in nurse practitioner curricula

Lack of expertise on faculty was considered to be a substantial barrier to including medical cannabis content in NP curricula by the majority of respondents (50%). The lack of evidence related to medical cannabis and not having medical cannabis as part of NPs’ scope of practice in most provinces/territories at the time of the study were also perceived by 40% of respondents to be significant barriers. See Table 3 for further details.

**Table 3:** Barriers to including Medical Cannabis in Curricula (*n* = 10)

Items	Frequency (%)^a^
Lack of expertise on faculty	5 (50.0)
Lack of evidence related to medical cannabis	4 (40.0)
Not part of nurses’/advanced practice nurses’ scope of practice	4 (40.0)
No space within the existing curriculum	2 (20.0)
Concerns about the safety of medical cannabis	3 (30.0)
Negative attitudes towards medical cannabis	1 (10.0)
Medical cannabis education available elsewhere for nurses	2 (20.0)
Other	2 (20.0)

^a^Respondents could select more than one option

### Beliefs regarding authority to authorize medical cannabis

All of the respondents indicated that specialist physicians (100%) should be authorized to approve the use of medical cannabis in Canada. The majority also supported NPs being able to authorize the use of medical cannabis (87.5%). See Table 4 for more information.

**Table 4:** Beliefs regarding Prescriptive/Authorization Ability (*n* = 8^a^)

Items	Frequency^b^ (%)
Specialist physicians	8 (100.0)
Primary care physicians/family physicians	7 (87.5)
Nurse practitioners	7 (87.5)
Nurses	3 (37.5)
Pharmacists	5 (62.5)
Naturopathic doctors	3 (37.5)
Traditional Chinese medicine practitioners	3 (37.5)
Others (i.e., Registered psychologists, NPs with special training)	4 (50.0)

^a^2 respondents failed to complete this section of the survey

^b^Respondents could select more than one option

## Discussion

As a growing number of jurisdictions around the world legalize cannabis for therapeutic purposes, nurses will be required to provide care to patients who are using or interested in medical cannabis. Governments, regulatory bodies, and healthcare institutions will need to develop policies to regulate nursing practice related to medical cannabis and ensure safe care is provided. Nursing education programs will also be faced with updating their curricula to provide the necessary knowledge and skills to address this nascent area of health care. To the best of our knowledge, this study is the first to examine nursing policies and education programs specific to medical cannabis. Although focused on medical cannabis in Canada, the findings highlight the nursing practice and policy issues that may exist in other countries.

### Medical cannabis policy and regulations

In our review of policy documents, it was striking that despite the establishment of a Canadian medical cannabis program in 2001, as well as the legalization of non-medical cannabis in 2018, not all nursing regulatory bodies had developed position statements, scopes of practice or regulations specific to cannabis. For those that had, there were some inconsistencies across jurisdictions regarding where nurses were allowed to administer medical cannabis (i.e., hospital versus community) and if they were able to directly administer or only assist in administration to patients. Where nurses were allowed to provide care related to medical cannabis, they were required to first consult with available institutional policies, which in some cases, could limit their ability to directly or indirectly administer cannabis to a patient.

The practice consultants interviewed in this study also discussed how these inconsistencies across federal, provincial/territorial and institutional policies created much confusion among nurses as well as for their employers. Greater clarity was needed regarding how cannabis is handled and disposed of, the amount of cannabis allowed to be possessed by a nurse, the distinction between direct versus assisted administration and the clinical settings in which cannabis could be administered by a nurse. Without these issues being addressed, care inequities for patients authorized to use medical cannabis will potentially exist across jurisdictions and care settings. Recently, the CNPS attempted to clarify the practice issues surrounding medical cannabis for NPs and registered and licensed practical nurses given the new Cannabis Act and Regulations [11]; however, the ambiguity between governmental policies, regulatory bodies’ standards, and institutional policies still remain.

It was also surprising that despite NPs being given the federal authority to authorize medical cannabis in 2016 [1], only one province had moved forward at the time of the study with including authorization within NPs’ scope of practice. This disconnect was most likely a consequence of cannabis being originally excluded under Health Canada’s New Classes of Practitioners Regulations, which were introduced in 2012 to expand the type of practitioners able to authorize and/or administer controlled substances [12]. Despite a recent amendment that permits NPs to authorize medical cannabis if the province/territory in which they practice includes medical cannabis within their regulations, cannabis has not yet been addressed within the educational competencies developed by the Canadian Association of Schools of Nursing (CASN) for the prescribing of controlled drugs and substances [13]. Without such competencies, it is unlikely medical cannabis content will be included in what are already packed NP curricula.

Since this study began, however, most of the nursing regulatory bodies in Canada have revised their policies (with the exception of Alberta and Quebec) to allow NPs to authorize medical cannabis. In addition, several regulatory bodies have developed practice statements regarding the possession and administration of medical cannabis. It will be important as federal cannabis regulations are revised in the future that nursing regulatory bodies be proactive in revising their policies in a timely manner and reduce the time lag between federal and provincial/territorial policy development and changes to nursing regulations.

An additional factor that may influence the inclusion of medical cannabis within NPs’ scope of practice is the recent recommendation by the Canadian Medical Association (CMA) [14] to eliminate the medical cannabis program following the legalization of non-medical cannabis and removing physicians as the gate-keeper to medical cannabis. This recommendation may leave a significant gap in the Canadian healthcare system that NPs could fill to ensure patients receive evidence-based access and care related to medical cannabis.

### Practice issues related to medical cannabis

Medical cannabis is challenging for nurses. As a natural substance, cannabis does not conform to traditional notions of medication with regards to production, standardization, drug administration, and dose. While general medication administration standards provide a good starting point for nurses involved in the administration of medical cannabis, specific guidelines will be needed to address the unique characteristics of cannabis use. This will include self-titration, administration of cannabis, interaction with medication, safety issues for both patients and healthcare providers (e.g., second-hand smoke exposure), and appropriate disposal of used material [15].

There is an urgent need for education and training related to medical and non-medical cannabis so that nurses have the necessary knowledge and skills to support patients in making informed decisions. Such education will also need to address the biases some nurses may hold regarding cannabis as a controlled substance versus a medicine [8]. Similar to other controversial issues in health care (e.g., medical assistance in dying), nurses caring for patients using medical cannabis will need to draw on their codes of ethics and practice standards in providing care that is respectful, non-judgmental, and evidence-informed. Conscientious objection by nurses who are unable to administer or authorize medical cannabis due to personal beliefs will need to be addressed in regulatory and institutional policies.

The legalization of non-medical cannabis in Canada will present some unique challenges for nurses, who may be confronted with patients who are using cannabis for both therapeutic recreational purposes [16]. Nurses will be expected to assess and provide care to individuals who may be experiencing problematic use, including cannabis use disorder [17]. Understanding and being able to enact guidelines, such as the Canadian Lower-Risk Cannabis Use Guidelines [18] will be important for all nurses to promote safe cannabis use and provide individuals with strategies to limit harm.

An additional practice issue posed by the legalization of non-medical cannabis is the possibility that some nurses may choose to legally use cannabis for recreational purposes. It will be vital that nurses, regulatory bodies, employers, workplace safety and occupational health specialists, and insurers come to a consensus on the concept of fitness to practice in light of legal non-medical cannabis. Fitness to practice is addressed within the Canadian Nurses Association’s Code of Ethics [19] and will be an important starting point in addressing nurses’ use of cannabis.

### Nurse practitioner programs and cannabis education

With the growing use of both medical and non-medical cannabis in Canada [19], NPs will be faced with an increasing number of Canadians either using or interested in learning more about cannabis. It will be imperative that NP programs in Canada include educational content on cannabis in their curriculum. NPs will need knowledge regarding current federal and provincial/territorial laws and regulations surrounding cannabis, as well as the latest research on the potential risks and benefits of cannabis use at an individual and community level. In addition, as research develops on the therapeutic use of cannabis, NPs will require education on appropriate treatment plans, dosing strategies, and administration protocols. Incorporating a harm reduction lens that includes key messages from Canada’s Lower Risk Cannabis Use Guidelines [18] across cannabis curricula would also be of value to NPs to reduce the potential risks associated with cannabis.

Given the expanded role of NPs in primary care as well as the growing number of Canadian nursing regulatory bodies including medical cannabis authorization within NPs’ scope of practice, it was surprising that just half of the NP programs participating in this study addressed the therapeutic benefits and risks of medical cannabis within their curricula and only one program included content on dosing and treatment plans. This is particularly striking when the majority of NP program coordinators believed that NPs should be able to authorize medical cannabis to eligible patients. NP programs need to be proactive in offering training and education on medical cannabis to their students in order to prepare them for the growing interest by patients as well as anticipated regulatory changes that will allow all NPs to authorize medical cannabis across Canada. Continuing education will also be urgently needed for those NPs already in practice and to address the developing body of evidence related to medical cannabis.

Several significant barriers will need to be addressed to facilitate the inclusion of cannabis content in NP curricula. Foremost, the lack of expertise among NP faculty in Canada will require a concerted effort by post-secondary institutions to support their faculty members in gaining the necessary knowledge and skills related to cannabis. On-line training programs specific to nurses have begun to be developed and would provide faculty with the foundational knowledge required to develop introductory content on cannabis. Self-study opportunities could also be a means through which NP faculty address their lack of knowledge.

While evidence on medical cannabis is in its nasceny, it is a rapidly evolving area of healthcare research [20] that NPs must be knowledgeable about to inform their practice. Clinical guidelines are beginning to be developed [18, 21] and will provide an important starting point in educating NPs about such concepts as self-titration, safer routes of administration, and minimizing side effects of cannabis use. In addition, the growing number of clinical trials on cannabis and cannabinoids also provide preliminary direction in terms of dosing and treatment plans [22] that may help guide NPs caring for patients interested in using medical cannabis.

Several limitations in this study must be acknowledged. Foremost, we did not have full participation across all nursing regulatory bodies and NP programs in Canada. However, we achieved geographical representation across Canada with regards to the practice consultants interviewed and NP programs surveyed. In addition, there has been an attempt in Ontario to standardize the curricula across nine primary care NP programs, one of which was included in our study. Thus, our findings regarding the inclusion of medical cannabis in NP curricula may reflect a larger number of programs. Finally, although this study was focused on Canadian nursing policy and experiences related to cannabis, it provides insights regarding the challenges that nursing regulatory bodies and education programs may face as a growing number of jurisdictions around the world legalize both medical and non-medical cannabis.

## Conclusions

Canadian nursing regulatory bodies must be proactive in developing policies and educational resources for nurses that will support them in providing safe and informed care related to cannabis. There is an urgent need for practice statements that provide guidance to nurses, particularly with regards to the administration of cannabis in hospital and community settings, as well as how to address requests for information from patients and family members. Harmonized medical cannabis regulations and policies at the federal, provincial/territorial and institutional level on what registered nurses and NPs can and cannot do from a practice and legal perspective regarding the administration and authorization of medical cannabis is required to ensure Canadians receive consistent and safe care from nurses. It is unavoidable that nurses will be caring for individuals who are using cannabis for therapeutic purposes – they will need clear direction and support from their education programs and regulatory bodies as well as clinical institutions to do so in ways that will promote patient well-being as well as protect the professionalism of nurses.

## Supplementary information

**Additional file 1.** Inteview Guide and OnlineSurvey.

## Data Availability

The datasets generated and/or analysed during the current study are not publicly available due to ethical reasons (to maintain participants’ anonymity and confidentiality) but are available from the corresponding author on reasonable request.

## Abbreviations

ACMPR: Access to cannabis for medical purposes regulations
CNA: Canadian Nurses Association
CNPS: Canadian Nurses Protective Society
MMAR: Marihuana medical access regulations
NP: Nurse practitioner
