# Supplementary material for: “Guarding their practice”: a descriptive study of Canadian nursing policies and education related to medical cannabis
Source: BMC Nurs. 2019 Dec 9;18:66. doi: 10.1186/s12912-019-0390-7 (PMC6902327; doi:10.1186/s12912-019-0390-7)
Supplement: Supplementary file 1 — Additional file 1. Inteview Guide and OnlineSurvey. [file 12912_2019_390_MOESM1_ESM.docx]

**Additional file 1 – Interview Guide**

1. What are the current policies at your college/association related to medical cannabis and registered nurses?

- Does the college/association have any practice statements?
- Is medical cannabis included in the nursing scope of practice? Competencies?
- Is there any required/recommended education or training? (i.e., required education on controlled substances, including cannabis?)

1. What are the current policies regarding medical cannabis for nurse practitioners in your province/territory?
   - If none, what are the future plans to address the role of nurse practitioner related to medical cannabis? What is the timeline for developing new policies?
2. How will these new policies be developed? And who will be involved in this process?
3. Are there any additional future plans regarding policies and/or practice statements related to cannabis and nurses?

- Policies?
- Scope of practice?
- Practice statements?
- Competencies?
- Prescription authority?
- Required/recommended education or training?

1. What are some of the potential concerns/challenges/ or barriers about nurses engaging in care related to cannabis?
   - Lack of policies
   - Education/training
   - Current level of evidence related to medical cannabis
   - Canadian Nurse Protective Society (CNPS) article published in January 2017 in the Canadian Nurse. Did you hear about the statement, which advised nurses not to administer cannabis to patients, despite an order from an MD/NP? If yes, did it influence the policies in your college?
   - Legal
   - Institutional barriers
   - Interprofessional care issues (i.e., conflict among MD/NPs, disclosure issues)
2. What are some of the facilitators regarding nurses engaging in care related to cannabis?
   - Current gaps in care
   - Support of institutions and patient advocacy groups
   - MDs’ hesitancy to engage in care related to medical cannabis
   - Increased need for harm reduction
3. What do you see as being some of the new opportunities, if any, for nurses related to medical cannabis?
   - New area of practice (i.e., cannabis nurse)
   - Employment opportunities through licensed producers of cannabis
   - Developing their own licensed production of medical cannabis and related products/services
4. What do you see as being the major practice issues for nurses related to medical cannabis?
5. What major practice issues related to medical cannabis have you had to deal with as a practice consultant?
   - Without providing any identifying information, could you share a case about a practice issue related to medical cannabis that a nurse has requested your advice on?
6. How do you perceive the future legalization of non-medical/recreational cannabis in Canada will impact the nursing profession and its practice?
   - Impact on medical cannabis?
   - Impact on Canadian health care system?
   - Education/training needs of nurses?
   - Impact on the role of public health/primary care nurses? Other nursing specialities?
   - Impact on nurses’ fitness to practice if consuming non-medical cannabis?
   - Other impacts?
7. Is there anything else related to nursing practice and medical cannabis that you would like to share with us?

**Additional file 1 – Online Survey – NP Program Coordinators**

**Cannabis Education in Nursing Programs Survey**

1. Is material about medical cannabis present in your undergraduate nursing curriculum?

____ Yes (**If Yes, go to Question 2**)

____ No (**If No, go to Question 4**)

____ Unsure

1. What course(s) include(s) material on medical cannabis in the undergraduate nursing program? Please provide the name of all courses in which this material is present:

________________________________________________________________________________________________________________________________________________

1. Which of the following content areas related to medical cannabis are included in your undergraduate nursing curriculum? Please select all that apply:

_____ Dosing and creating effective treatment plans for patients using medical cannabis

_____ Similarities and differences between dried cannabis, other forms of cannabis products, and prescription cannabinoid medications

_____ Health Canada’s Access to Cannabis for Medical Purposes Regulations Program

(ACMPR)

_____ Laws and regulations surrounding the medical use of cannabis in Canada

_____ Safety, warning signs and precautions for patients using medical cannabis

_____ Alternative routes of administration of medical cannabis

_____ Mechanism of action of cannabis (endocannabinoid system)

_____ Potential risks of using cannabis for medical purposes

_____ Potential therapeutic uses for cannabis

_____ Other (please specify):______________________________

1. Is material about medical cannabis present in your nurse practitioner/advanced practice nursing curriculum?

____ Yes (**If Yes, go to Question 5**)

____ No (**If No, go to Question 7**)

____ Unsure

1. What course(s) include(s) material on medical cannabis in the nurse practitioner/advanced practice nursing program? Please provide the name of all courses in which this material is present:

________________________________________________________________________

________________________________________________________________________

1. Which of the following content areas related to medical cannabis are included in your nurse practitioner/advanced practice nursing curriculum. Please select all that apply:

_____ Dosing and creating effective treatment plans for patients using medical cannabis

_____ Similarities and differences between dried cannabis, other forms of cannabis

products, and prescription cannabinoid medications

_____ Health Canada’s Access to Cannabis for Medical Purposes Regulations Program

_____ Laws and regulations surrounding the medical use of cannabis in Canada

_____ Safety, warning signs and precautions for patients using medical cannabis

_____ Alternative routes of administration of medical cannabis

_____ Mechanism of action of cannabis (endocannabinoid system)

_____ Potential risks of using cannabis for medical purposes

_____ Potential therapeutic uses for cannabis

_____ Other (please specify):

__________________________________________________________________

1. What have been some of the barriers, if any, your nursing school has experienced regarding including content about medical cannabis in the curricula? Please indicate all that apply.

_____ Lack of expertise on faculty

_____ Lack of evidence related to medical cannabis

_____ Not part of nurses’/advanced practice nurses’ scope of practice

_____ No space within the existing curriculum

_____ Concerns about the safety of medical cannabis

_____ Negative attitudes towards medical cannabis

_____ Medical cannabis education available elsewhere for nurses

_____ No barriers have been experienced

_____ Other (please specify): ____________________________________

1. Are there any future plans to include content about medical cannabis in your undergraduate nursing program?

____ Yes

____ No

____ Unsure

1. Are there any future plans to include content about medical cannabis in your nurse practitioner/advanced practice nursing program?

____ Yes

____ No

____ Unsure

1. In your opinion, which health professionals should have the authority to authorize/prescribe medical cannabis?

Yes No

Specialist physicians ___ ___

Primary care physicians/family physicians ___ ___

Nurse practitioners ___ ___

Nurses ___ ___

Pharmacists ___ ___

Naturopathic doctors ___ ___

Traditional Chinese medicine practitioners ___ ___

Others (please specify): ___ ___

___________________________________

1. Is there anything else you would like to share about medical cannabis education in nursing programs in Canada?

________________________________________________________________________________________________________________________________________________

**Thank you for taking the time to complete this survey**
